# Silica Aerogel-Modified Polyacrylonitrile Nanofibers to Reduce Heat Flux in Heat Storage Tanks of Greenhouse Buildings

**DOI:** 10.3390/polym16152219

**Published:** 2024-08-03

**Authors:** Yuze Li, Yongping Zhang, Wenbo Sun

**Affiliations:** 1 The High School Affiliated to Renmin University of China, Beijing 100080, China; 2 Department of Physics, Tsinghua University, Beijing 100084, China

**Keywords:** greenhouse building, heat storage tank, heat flux, polyacrylonitrile nanofibers

## Abstract

Polyacrylonitrile (PAN) nanofibers have specific characteristics such as thermal insulation, weatherproofing, and sunlight resistance and therefore are appropriate to be applied as insulation materials for various industries, especially in greenhouse construction. The heat source in greenhouse buildings that operate independently in the heating network comes from heat storage tanks. In the present study, employing thermal field numerical simulations, we investigate the heat flux of a cylindrical heat storage tank with silica aerogel-modified PAN nanofibers as thermal insulation materials. The geometric scale of the tank body, thermal insulation material thickness, and outdoor temperature are optimized to improve thermal insulation. The significant discrepancy in heat flux at different parts of the heat storage tank leads to the extreme heat flux arising at the water–gas interface on the inner and outer walls. It is indicated that the heat flux distribution can be effectively ameliorated by modifying the scale of the tank body to retain the overall water temperature. In particular, effective insulation can merely be acquired when the thermal conductivity of the insulation material is below 3.3 W·m^−1^·K^−1^. Eventually, the heat storage tank is optimized to store 1400 L water at 100 °C with a radius of 0.6 m and a thermal insulation thickness of 50 mm at an outdoor temperature of −10 °C, which can maintain excellent thermal insulation for 8 and 24 h at 87.7 and 69.9 °C, respectively.

## 1. Introduction

Polyacrylonitrile (PAN) nanofibers are macromolecular polymers formed by the free radical polymerization of monomer acrylonitrile [1]. It has many advantages, mainly excellent heat retention, due to its porous fiber structure, which allows it to effectively keep air inside and provide a good insulation effect; good elasticity, meaning that it is able to maintain a high rebound rate even after stretching; strong weather resistance; good resistance to UV radiation and climate effects; ability to maintain relatively stable physical properties even after long-term exposure to outdoor environments; and excellent chemical resistance, with good resistance to many inorganic acids, bases, and some organic solvents [2,3,4,5]. PAN nanofibers are often used as thermal insulation materials in various industries such as textiles and construction [6,7].

The concept of zero carbon or low-carbon emissions in greenhouse buildings has the advantages of low energy consumption and environmental friendliness, which can be achieved in both large and individual buildings, with good flexibility [8,9,10,11,12]. The main function of greenhouse buildings is to achieve indoor temperature control, and the heat source is the core component of temperature control [13,14,15]. Independently operated greenhouse buildings rely on clean energy sources such as solar and geothermal energy for heating, and thermal energy is stored through heat storage thanks to the regulation of indoor temperature [16,17,18]. Using PAN nanofibers as thermal insulation material for heat storage tanks is a reliable choice. However, there is still a significant difference in the thermal insulation requirements of PAN nanofibers for heat storage tanks. PAN nanofibers were improved by using silica aerogel (SA) to reduce the thermal conductivity of materials [2]. The specific principle is that aerogel is similar to the glue matrix, while glass fiber is similar to the skeleton to form a composite thermal insulation material with certain strength and thermal insulation effect [19,20,21,22]. The thermal storage tank should be optimized and designed based on the building scale and environmental temperature, in order to achieve a good insulation effect at a low cost as much as possible [23,24].

In the present study, we propose silica aerogel (SA)-modified PAN nanofiber as the thermal insulation material to optimize temperature distribution and reduce heat flux in the cylindrical heat storage tank of independent operating greenhouses. The study is conducted on the insulation effect of 1400 L underwater with a storage tank capacity of 1500 L and a storage temperature of 100 °C, focusing on the influence of PAN nanofibers with different modification conditions on the thermal performance of heat storage tanks, the cylinder radius dependence of surface heat flux in the heat storage tank for optimizing the overall heat flux, the adjustment of insulation layer thickness in the heat storage tank to enhance the thermal insulation effect with less thermal insulation material, and the influence of environmental temperature on refrigeration power to elucidate the relationship between environmental temperature and the thermal performance of heat storage tanks.

## 2. Numerical Simulation Methodology

**Figure 1:**
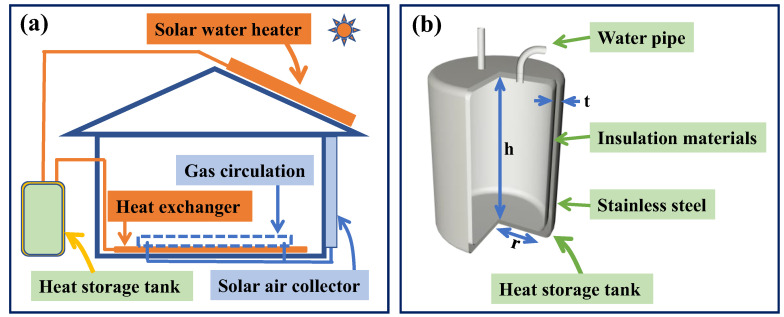
Schematic diagram of model structures: (**a**) temperature control system in greenhouse independent of the heat network; (**b**) internal structure of cylinder heat storage tank.

The numerical simulations of temperature and heat flux distributions in the heat storage tank, as schematically shown in Figure 1, were carried out by the heat transfer module of COMSOL 6.0 software. There are two ways for a water medium and an air medium to control the indoor temperature. Regulating indoor temperature through an air medium typically occurs under conditions of intense solar radiation and long daylight hours, such as during the sunny afternoons of summer. Conversely, regulating indoor temperature through a water medium is more suitable under conditions where solar radiation is relatively insufficient or limited, such as on cloudy or overcast days, or during the early mornings and late evenings of winter. The external environment of the heat storage tank, such as temperature and atmospheric pressure, was set up. During the process of adding materials, water with an initial temperature of 100 °C was added. The area containing water was configured as an isothermal domain. In this work, the total volume and the water volume in the heat storage tank were specified to be 1500 L and 1400 L, respectively. The temperature change for 24 h and the cooling power of the top and side of the heat storage tank were analyzed. Under the condition that the volume (*V*) of the heat storage tank remained constant, the geometric structure was optimized by changing the radius (*r*) and height (*h*) coordinately to further enhance the temperature and heat flux distribution. The heat storage tank was made of stainless steel (Steel AISI 4340) with a double-layer structure, and the middle of a double-layer shell was made of thermal insulation material, as shown in Figure 1b. In the numerical simulations of heat transfer in the heat storage tank, the heat capacity of thermal insulation materials was set to 200 J·m^−3^·K^−1^, which was almost equal for both PAN and SA, while the thermal conductivities of thermal insulation materials were specified according to Table 1 [2].

## 3. Results and Discussion

### 3.1. Modification of Polyacrylonitrile Nanofiber

**Figure 2:**
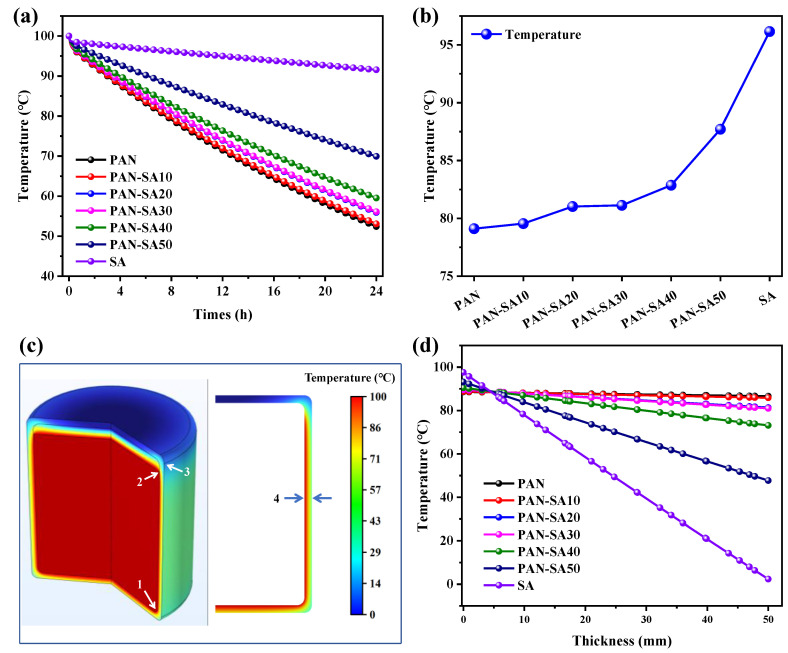
The thermal performance of the heat storage tank varies with different insulation materials: (**a**) water temperature evolution in heat storage tank over time within 24 h; (**b**) water temperatures inside heat storage tanks with different insulation materials at the 8th hour; (**c**) temperature distribution of water and wall in heat storage tank with PAN-SA50 at the 1st hour; (**d**) temperature distribution at arrow 4 in tank wall at the 1st hour.

When setting the thickness of the thermal insulation material to 50 mm and the radius of the heat storage tank to 0.6 m, the thermal performance of heat storage tanks with different thermal insulation materials was evaluated by temperature evolution and distribution in water and walls, as shown in Figure 2. Within the range of 0 to 0.1 h, the temperature of water decreased rapidly. Over time, the temperature evolution shows a linear downward trend. The rate of temperature reduction varied for different insulation materials. As the mix concentration increased, the thermal conductivity gradually decreased, and the rate of temperature decrease also slowed down. When the thermal conductivity of thermal insulation material was higher than 3.3 W·m^−1^·K^−1^, the decrease in water temperature was almost the same for different thermal conductivities, implying poor heat insulation. When the thermal conductivity was below 3.3 W·m^−1^·K^−1^, the rate of water temperature decrease gradually slowed down, indicating the enhancement of heat insulation from the thermal insulation layer. The thermal conductivity of PAN-SA50 was 0.4 W·m^−1^·K^−1^, and the water temperature could still be maintained at 87.7 °C at the 8th hour and 69.9 °C at the 24th hour. The thermal conductivity of PAN was 15.8 W·m^−1^·K^−1^, and the water temperature could still be maintained at 79.1 °C at the 8th hour and 52.4 °C at the 24th hour. The water temperature of doped PAN was significantly higher than that of undoped PAN.

As shown in Figure 2b for the dependence of water temperature on thermal insulation materials at the 8th hour, the water temperature approached the lowest value when using PAN, which was increased, however, by increasing the concentration of SA in the PAN thermal insulation layer. In comparison, PAN-SA50 had the highest water temperature compared with other SA mix concentrations. In order to have a clearer understanding of the impact of different materials on the thermal performance of heat storage tanks, the temperature distribution of the tank at the first hour and the temperature gradient of the tank wall were calculated. Three positions are marked on the side of the heat storage tank for a clear representation of the thermal properties of the tank walls. Figure 2c shows that the temperature of the air in the heat storage tank is relatively lower, and the temperature at the top of the tank wall is much lower than that on the side and ground. In comparison, the temperature change on the side of the heat storage tank wall was more significant at the junction of water and air, while the temperature in direct contact with water was relatively higher. Overall, the temperature at the bottom of the heat storage tank wall was much higher than other parts, verifying that the bottom and sides were the dominant areas for heat release.

As indicated by Figure 2d for the temperature change in the tank wall from the inside out at the position of arrow 4, labeled in Figure 2c, the temperatures of the tank walls with PAN and the thermal insulation materials with lower mix concentrations remained almost unchanged, implying poor thermal insulation performance. As the mix concentration increased, the temperature on the interior side was lower than that on the exterior side of the tank wall, implying that the thermal insulation material acquired a higher surface thermal insulation.

**Figure 3:**
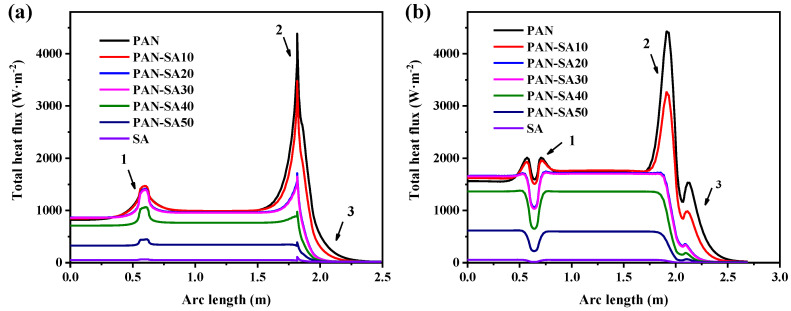
The heat flux (**a**) inside and (**b**) outside the walls of the heat storage tank at the 0.1th hour, where the arc length represents the length from the bottom center point through the bottom, side, and top edges to the top center point.

In order to provide more detailed proof of the influence of different thermal insulation materials on the thermal performance of the heat storage tank, the heat fluxes of the inner and outer walls of the heat storage tank were calculated separately, as shown in Figure 3. The horizontal axis in the figure represents the arc length, starting from the center of the bottom surface of the heat storage tank and passing through the side, reaching the center of the top edge. The entire arc length is illustrated on the right in Figure 2c. As shown in Figure 3a for the heat flux on the inner wall of the heat storage tank, PAN with the highest thermal conductivity yielded the highest heat flux on the inner wall. After mixing with SA, the heat flux of the inner wall gradually decreased due to a decrease in the thermal conductivity of PAN with an increase in the SA concentration, which was also verified by the smallest heat flux on the inner wall using pure SA as the thermal insulation layer. The heat flux of the inner wall increased at the junction of the bottom and the side. At the junction of water and air, the heat flux of the surface was the highest, and there was a certain temperature change, while the heat flux at the top of the heat storage tank was the lowest. As indicated in Figure 3b, the heat flux outside the walls of the heat storage tank was directly determined by the thermal conductivity of the thermal insulation material. The heat flux of the outer wall was the smallest at the junction of the bottom and side edges, and the insulation material here was thicker compared to other positions. Meanwhile, a higher heat transfer coefficient of thermal insulation material led to a higher heat flux at the water–air interface. Therefore, it was also required that the upper limit of the thermal conductivity of the thermal insulation material used in the heat storage tank adequately maintain a small heat flux at the water–air interface.

**Figure 4:**
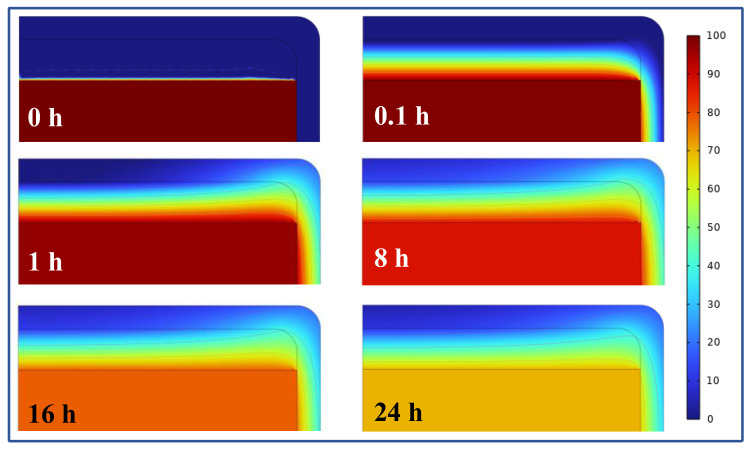
Temperature distribution from 0 °C to 100 °C in the heat storage tank for different heat release times.

There was a sudden temperature change at the interface between water and air in the heat storage tank, as shown in Figure 3a. Thereby, PAN-SA50 was chosen as the thermal insulation material to improve the temperature distribution at different times at this location, as shown in Figure 4. At the 0th hour, there was a significant difference in temperature between water, air, and the walls of the heat storage tank. This occurred during the initial trial period when the heat had not yet been exchanged. At the 0.1th hour, a temperature gradient appeared at the interface between water and air, and heat was exchanged between water and air. The farther the temperature in the air was from that of the water surface, the lower the temperature. This also indicated that the top of the heat storage tank wall was separated by air, resulting in a lower heat flux. On the side wall of the heat storage tank, there was a significant temperature change at the junction of water and air. The side wall temperature of the heat storage tank in contact with air was lower, while the side wall temperature of the heat storage tank in contact with water was higher. Over time, the isotherm in this region shifted upwards, and the flow of heat also caused the temperature of water to continue to decrease. It should be noted that although SA has relatively the highest heat resistance, its poor mechanical properties, such as brittleness and easy breakage or crushing, make it unsuitable for use as a thermal insulation material for heat storage tanks. Therefore, PAN-SA50 is preferred for use as a thermal insulation material for improving the thermal performance of heat storage tanks.

### 3.2. Geometry Optimization of the Heat Storage Tank

**Figure 5:**
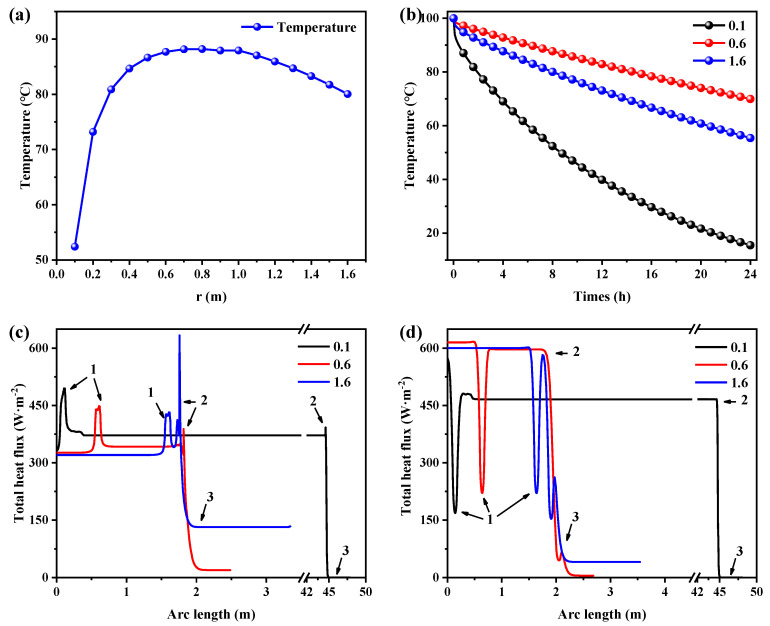
Thermal performance of the heat storage tanks varying with tank radius when using PAN-SA50 as the thermal insulation material: (**a**) water temperatures in the heat storage tank as a function of tank radius at the 8th hour; (**b**) temperature evolution starts at 100 °C with time for different tank radii; the heat flux (**c**) inside and (**d**) outside the walls of the heat storage tank at the 0.1th hour, where the arc length represents the length from the bottom center point through the bottom, side, and top edges to the top center point.

It is indicated from Figure 3 that different positions on the wall of the heat storage tank have different heat loss rates, which results in different degrees of thermal performance of the heat storage tank with different geometric parameters. With a constant volume of 1500 L, PAN-SA50 was selected as the thermal insulation material with a thickness of 50 mm, and the outdoor temperature was set to −10 °C. Figure 5a shows the relationship between the water temperature and the radius of the heat storage tank after 8 h when storing 1400 L at a temperature of 100 °C. As the radius increased, the water temperature first increased and then decreased. At a radius of 0.6 to 1.0 m, the peak temperature was reached, indicating that the overall heat flux was the smallest at that radius. Figure 5b shows the relationship between the water temperature and time at radii of 0.1 m, 0.6 m, and 1.6 m, respectively. At all radii, the water temperature decreased rapidly at the beginning and then gradually decreased, indicating that the overall heat dissipation power of the heat storage tank at different times is related to the temperature of the water. In all time periods, with the increase in radius, the water temperature also showed a law of first increasing and then decreasing. Therefore, the optimal radius was 0.6 to 1.0 m.

The heat fluxes on the surfaces of the heat storage tank considerably rely on its geometry, as indicated by Figure 5c,d, which show the heat flux distributions on the bottom, side, and top edges of the inner and outer walls of the heat storage tanks with various radii at 0.1 h. When the radius was 0.1 m, the side area of the heat storage tank was the largest, the heat was mainly lost through the bottom and side edges, and the heat flux inside the heat storage tank wall was the highest at all positions. When the radius was 0.6, the side area and bottom area of the heat storage tank were relatively average, and the internal heat flux of the heat storage tank wall was relatively high, most of which occurred below the radius of 0.1 m. When the radius increased to 1.6 m, although the top area of the heat storage tank became larger, and the heat flux at the top was smaller, the area at the bottom was also very large, and the heat loss was greater. The maximum heat flux was at the junction of the side and bottom, and at the water–air interface; the larger the radius, the greater the heat flux at the interface between water and air. The heat flux outside the heat storage tank wall was different from that of the inside. The heat flux at the bottom and side edges of the heat storage tank was almost the same, and the heat flux at the contact between the bottom and side edges was the smallest. When the radius was 0.1, the heat flux was smaller than that of the other two radii. However, its side area was the largest, and the total heat loss was greater than the other two cases. When the radii were 0.6 and 1.6 m, the difference in the heat flux was not significant. However, due to different radii, the positions of the junction of the bottom and side surfaces and the contact between water and air surfaces are at different positions of the arc length, so the positions of the minimum heat flux are different. In summary, if the radius was too small, more heat was lost from the side, and the thermal performance of the heat storage tank was reduced. When the radius of the heat storage tank increased to 0.6 m, the heat storage tank had better thermal performance.

### 3.3. Optimization of Thermal Insulation Thickness

**Figure 6:**
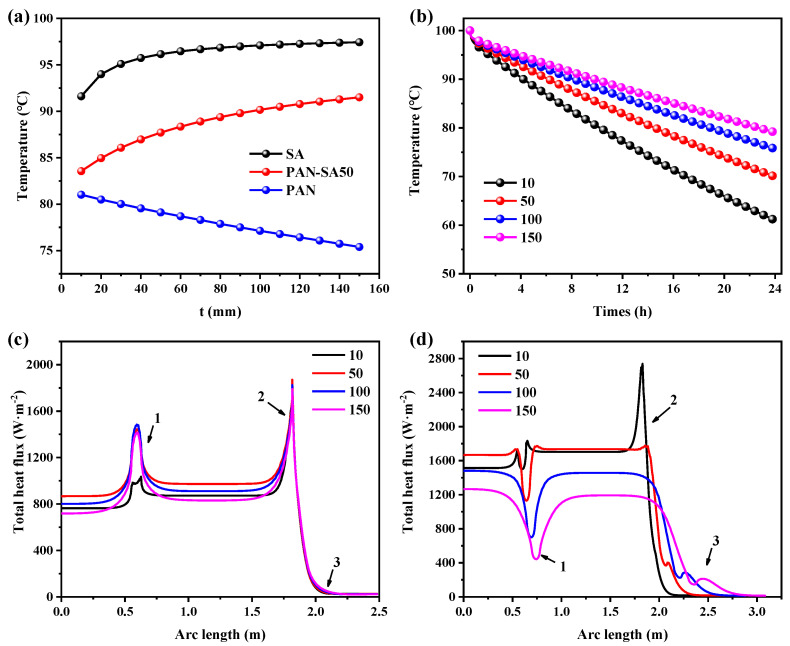
Thermal performance of heat storage tank in dependence on insulation layer thickness and thermal insulation material: (**a**) water temperature varying with insulation layer thickness at the 8th hour; t is the thickness of insulation materials; (**b**) water temperature changing with time when using PAN-SA50 material; the heat flux (**c**) inside and (**d**) outside the tank wall at the 0.1th hour, where the arc length represents the length from the bottom center point through bottom, side, and top edges to the top center point.

The thickness of thermal insulation material has a great influence on the heat flux and ultimately affects the thermal insulation effect. Here, the relationship between thermal insulation thickness and heat flux of the heat storage tank made of PAN, PAN-SA50, SA with a radius of 0.6 m and an outdoor temperature of −10 °C was studied. Figure 6a shows the change in water temperature in the heat storage tank with the thickness of thermal insulation material at the 8th hour. When the insulation materials were SA and PAN-SA50, the water temperature increased rapidly at first and then slowly with the increase in the thermal insulation thickness. When the thickness of PAN-SA50 thermal insulation material was 50 mm, the water temperature at the 8th hour was 87.7 °C, which was considered to be the temperature that allowed for better thermal insulation. In the beginning, radial heat conduction was the deciding factor; however, as the size of the object increased, radial heat conduction was no longer the main factor. The surface area became the key factor that determined the speed of heat dissipation. However, due to the higher thermal conductivity of PAN compared with PAN-SA50 and SA, the water temperature continued to decrease with the increase in thermal insulation thickness, indicating the poor thermal insulation effect of using PAN. Figure 6b shows the changing trend of water temperature in the heat storage tank under different thermal insulation thicknesses as a function of heat release time when using PAN-SA50 as the insulation material. With the increase in the thickness of the thermal insulation layer, the overall temperature increased, and the increased amplitude gradually weakened. At the 8th hour, the water temperatures at thicknesses of 50 mm, 100 mm, and 150 mm showed slight differences.

Figure 6c,d, respectively, show the heat flux of the inner and outer walls of the heat storage tank under different insulation thicknesses. With the increase in the thickness of the insulation material, the heat flux of the inner wall changed little, while the heat flux of the outer wall decreased gradually, and the decreasing range gradually slowed down, consistent with the previous temperature change law. Here, the temperature of the inner and outer walls of the heat storage tank was calculated after 8 h when the thickness of the thermal insulation material was 50 mm, as shown in Figure 2c. The temperature of the heat storage tank wall with different thermal insulation thicknesses at the location marked as arrow 4 in Figure 2c was calculated, as shown in Figure 2d. It was found that with the increase in the thickness, the temperature of the thermal insulation layer inside the double-layer wall of the heat storage tank was the same and increased with the increase in the thickness. This shows that with the increase in the thickness of the insulation layer, heat is more difficult to transfer to the external environment. Finally, it should be highlighted that when the thickness of the thermal insulation layer was 50 mm, a better effect was achieved. The effect of increasing the thickness of the thermal insulation layer is not significant, and it is not conducive to controlling the cost.

### 3.4. The Influence of Outdoor Temperature on Thermal Performance

**Figure 7:**
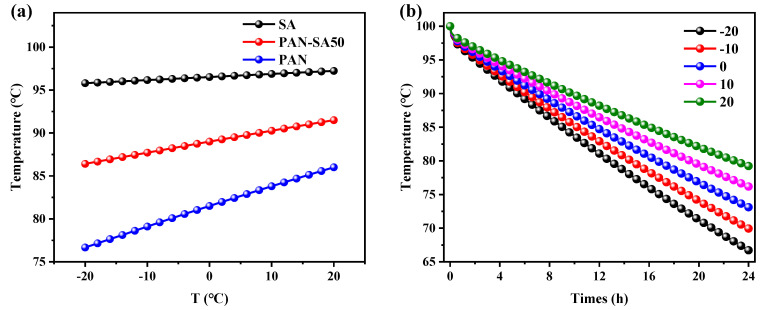
Water temperature in the heat storage tanks; T is the environmental temperature (**a**) varying with environmental temperature for various thermal insulation materials at the 8th hour and (**b**) as a function of heat release time for PAN-SA50 thermal insulation material at various environmental temperatures.

After optimizing the geometric scale and the thermal insulation thickness, the influence of outdoor (environmental) temperature on the refrigeration power was further studied, especially for the optimized heat storage tank with a tank radius of 0.6 m and a thermal insulation thickness of 50 mm. The water temperature in the heat storage tanks using three kinds of thermal insulation materials was calculated, with the results shown in Figure 7. It is demonstrated that the water temperature and environmental temperature exhibit a linear relationship. In comparison, the outdoor temperature had the minimum and maximum impact on the thermal performance of the heat storage tanks with SA and PAN thermal insulation layers, respectively, as shown in Figure 7b. Considering the water temperature evolution of the heat storage tank over time using PAN-SA50 as thermal insulation under various environmental temperatures, as shown in Figure 7b, it was verified that a reduction in environmental temperature evidently exacerbated heat release and therefore the water temperature retention of the heat storage tank.

## 4. Conclusions

Considering independently operated greenhouses and heat supply networks, we proposed utilizing SA-modified PAN nanofibers (SA-PAN) as thermal insulation materials for heat storage tanks and accordingly optimizing the structure of heat storage tanks. The effects of radius, thermal insulation material thickness, and environmental temperature on the thermal performance of a cylindrical heat storage tank with a capacity of 1500 L were analyzed for maximizing thermal insulation from SA-PAN. It was found that PAN-SA50 basically meets the short-term heat storage requirements of the heat storage tank. The geometric structure of the heat storage tank changed the heat transfer, resulting in different heat fluxes on the side and top. The 100 L volume of air above the heat storage tank caused the heat flux on the top surface to be lower than that on the bottom surface. After optimizing the heat flux of the top and the side, the overall heat flux was low, and the thermal insulation effect was significantly improved when the tank radius approached 0.6 mm. With the increase in the insulation layer thickness, the overall insulation effect showed a rapid upward trend. When the thickness was greater than 50 mm, the change in the thermal insulation effect gradually decreased. After optimization, the heat storage tank could maintain the temperature at 87.7 °C after 8 h and 69.9 °C after 24 h when it stored 1400 L of 100 °C water, the tank radius approached 0.6 m, and the thermal insulation thickness was specified as 50 mm at the outdoor temperature of −10 °C.

## Figures and Tables

**Table 1:** Thermal conductivities specified for thermal insulation materials in heat transfer numerical simulations of a heat storage tank.

Thermal Insulation Material	Thermal Conductivity (W·m^−1^·K^−1^)
PAN	15.8
PAN-SA10	10.6
PAN-SA20	3.5
PAN-SA30	3.3
PAN-SA40	1.5
PAN-SA50	0.4
SA	0.037

## Data Availability

The raw data supporting the conclusions of this article will be made available by the authors upon request.
